# *Streptomyces* sp. VITGV156 secondary metabolite binds pathogenic protein PBP2a and Beta-lactamase

**DOI:** 10.3389/fbinf.2025.1544800

**Published:** 2025-03-26

**Authors:** Veilumuthu Pattapulavar, Sathiyabama Ramanujam, Manisha Shah, Muthu Kumar Thirunavukkarasu, Sivakumar Arumugam, Ramanathan Karuppasamy, Antony V. Samrot, K. Deepasree, Subhashree Venugopal, John Godwin Christopher

**Affiliations:** ^1^ Department of Biomedical Sciences, School of BioSciences and Technology, Vellore Institute of Technology, Vellore, India; ^2^ Department of Science and Humanities, Karpagam Academy of Higher Education, Coimbatore, Tamil Nadu, India; ^3^ Department of Bio-Sciences, School of BioSciences and Technology, Vellore Institute of Technology, Vellore, India; ^4^ School of Sciences and Humanities, SR University, Warangal, Telangana, India; ^5^ Department of Biotechnology, School of BioSciences and Technology, Vellore Institute of Technology, Vellore, India; ^6^ Department of Microbiology, Faculty of Medicine, Manipal University College Malaysia, Melaka, Malaysia; ^7^ Department of Integrative Biology, School of BioSciences and Technology, Vellore Institute of Technology, Vellore, India

**Keywords:** *Streptomyces*, secondary metabolites, antiSMASH, terpenoids, molecular docking

## Abstract

**Introduction:**

The genus *Streptomyces* is renowned for its prolific production of bioactive compounds, including antibiotics and secondary metabolites with pharmaceutical applications. This study focuses on *Streptomyces* sp. VITGV156, an isolate with promising antimicrobial properties, aiming to characterize its genomic potential and bioactive compounds through computational and experimental analyses.

**Methods:**

Genomic sequencing of *Streptomyces* sp. VITGV156 was performed to identify biosynthetic gene clusters (BGCs) associated with secondary metabolite production. Antimicrobial assays were conducted using crude extracts against Gram-positive and Gram-negative pathogens. Gas Chromatography-Mass Spectrometry (GC-MS) was employed to identify secondary metabolites. Additionally, ADME (Absorption, Distribution, Metabolism, and Excretion) analysis and molecular docking studies were conducted to assess drug-like properties and binding affinities of selected compounds against bacterial target proteins (PDB IDs: 5M18 and 6NVU).

**Results:**

The genome of *Streptomyces* sp. VITGV156 was determined to be 8.18 Mb with a G+C content of 72.61%, containing 29 BGCs responsible for the biosynthesis of antimicrobial agents such as nystatin and fluostatins. In vitro antimicrobial assays confirmed strong efficacy of crude extracts against various pathogens, with *Escherichia coli* exhibiting the highest susceptibility. Molecular docking studies of 45 identified secondary metabolites revealed binding affinities ranging from -4.0 to -7.5 kcal/mol (5M18) and -3.9 to -7.2 kcal/mol (6NVU). Among the identified compounds, squalene (ligand 43) displayed potent antibacterial and antifungal activity, whereas 2,5-piperazinedione, 3-(hydroxymethyl)-6-(phenylmethyl)- (ligand 40) exhibited strong antifungal potential. Conversely, fumaric acid, monoamide, N-benzyl-N-phenylethyl-, ethyl ester (ligand 38) demonstrated weak antifungal activity.

**Discussion:**

The genomic and bioactive analysis of *Streptomyces* sp. VITGV156 highlights its potential as a valuable source of novel antimicrobial agents. The identification of unique biosynthetic genes and bioactive secondary metabolites suggests its possible application in combating multidrug-resistant pathogens. Further studies, including purification and in vivo testing, are necessary to validate these findings and explore their therapeutic potential

## 1 Introduction

The rise of microbial resistant pathogens has become a global challenge, necessitating the discovery and development of new antibiotics. *Streptomyces*, a genus of actinobacteria, has emerged as a leading source of bioactive secondary metabolites, particularly antibiotics. These organisms are credited with producing a significant proportion of clinically relevant antibiotics, which inhibit key cellular processes in pathogens. The versatility of *Streptomyces*-derived compounds lies in their ability to target specific bacterial proteins, often essential for survival, making them effective weapons against both Gram-positive and Gram-negative bacteria (Veilumuthu P. and Christopher G. J., 2022). These compounds belong to the group of antibiotics, antifungals, and anticancer agents, accounting for about 80% of naturally derived antibiotics in use today. Recent studies have identified over 279 new bioactive compounds from *Streptomyces* between 2015 and 2020, highlighting its ongoing relevance in combating antibiotic resistance and emerging diseases (Kiran et al., 2022).

*Streptomyces* are predominantly inhabiting soil and foster beneficial interactions with plants (Klykleung et al., 2020). These organisms play an important role by generating a spectrum of metabolites that enhance plant growth and protection. Their synergistic association with host plants underscores their significance as a reservoir of bioactive natural products. The strategic collection of varied *Streptomyces* strains holds the potential for unearthing novel natural products (Mishra et al., 2021; Ma and Karthik, 2022). Despite their significance in managing plant diseases, augmenting productivity, and influencing physiological activities, substantial aspects of their associations remain undiscovered (Kalyani et al., 2019; Ahmad and Zin, 2021).

As of November 2024, the genus *Streptomyces* includes 1,240 recognized species and 75 subspecies, classified within three genera. To date, 1,240 genomes of *Streptomyces* have been sequenced, with corresponding data entries cataloged in 12,150 SRA records available in the NCBI database. Moreover, computational tools facilitate the discovery of novel antibiotics by mining the genomes of *Streptomyces* species. Advanced algorithms identify biosynthetic gene clusters responsible for producing secondary metabolites, accelerating the identification of compounds with therapeutic potential. This integration of computational and experimental methods enables not only the prediction of novel bioactive molecules but also the optimization of existing ones to overcome resistance mechanisms. The ability of *Streptomyces* to produce diverse antibiotics, combined with the precision of computational biology, underscores their significance in addressing the global antibiotic crisis. By targeting specific bacterial proteins and processes, these compounds offer a targeted approach to combating infections. The continued exploration of *Streptomyces*-derived antibiotics and their mechanisms of action, supported by computational advances, holds promise for the development of next-generation therapeutics to mitigate the growing threat of antimicrobial resistance.

However, *Streptomyces* sp. stands as a prolific source of unexplored secondary metabolites, harbouring multiple biosynthetic gene clusters (BGCs) within its genome. Employing genome mining techniques has unveiled latent BGCs in *Streptomyces* genomes, underscoring their potential for novel drug discovery. Sequencing *Streptomyces* genomes principally aims to identify antibiotics characterized by unique mechanisms of action (Caicedo-Montoya et al., 2021). This investigation aims to analyze diverse secondary metabolites of *Streptomyces* sp. VITGV156 and its secondary metabolites that inhibit the growth of four different bacterial strains. Further whole genome sequencing was performed to explore the genome of *Streptomyces* sp. VITGV156. This study paves the way for the identification of compounds from *Streptomyces* sp. VITGV156, which can be exploited for commercially valuable secondary metabolites.

## 2 Materials methods

### 2.1 *Streptomyces* sp. VITGV156

A pure culture of the *Streptomyces* strain VITGV156 was sourced from the Microbiology Laboratory, SBST, VIT University, Vellore, and maintained at −20°C. To revive the strain, it was streaked on fresh International *Streptomyces* Project 2 (ISP2) agar medium, following the procedures described in our prior isolation and publication of this strain (Veilumuthu et al., 2024).

### 2.2 GC-MS analysis of crude extracts of *Streptomyces* sp. VITGV156

The *Streptomyces* sp. VITGV156 strain was cultured in ISP-2 medium with 1% under incubator with shaker for 21 days. It is to note that standard fermentation conditions were applied during the production process. During fermentation, pellet formation was observed in the clear culture broth. The culture supernatant was separated by centrifugation at 3,000 rpm for 10 min then the secondary metabolites was extracted with ethyl acetate (1:1, v/v) and analyzed in GC-MS. (Thermo Scientific system). The setup featured a Trace GC Ultra, ISQ Single Quadrupole MS, and a TG-5MS fused silica column (30 m × 0.25 mm × 0.1 mm film thickness). Detection employed electron ionization at 70 eV with helium as the carrier gas (1 mL/min). The temperature ranged from 70°C to 270°C at 4°C/min. Spectral comparison with the Wiley database identified metabolites (Veilumuthu and Godwin, 2022).

### 2.3 Assessment of antibacterial activity–well diffusion assay

The antimicrobial activity of the ethyl acetate extracts was determined by a well-diffusion assay (Magaldi et al., 2004; Valgas et al., 2007). Using sterile metal borers wells of 6 mm were dug into sterile LB agar plates. Four distinct bacterial strains, namely, *Escherichia coli* (MTCC11105), *S. aureus* (MTCC6571), *P. aeruginosa* (MTCC25619), and *B. subtilis* (MTCC3610) were inoculated in the Mueller Hinton agar (MHA) through swabbing. Ethyl acetate extracts of VITGV156 were added to the wells at a concentration of 25, 50, 75 and 100 μg/mL and incubated at 37°C for 24 h. The antimicrobial activity was quantified by measuring the inhibition zones (millimetres). The experiment was repeated thrice, and the average value was plotted with standard error mean using Graph pad Prism 9.0.

### 2.4 Genome characterization and annotation

Genomic DNA was extracted from *Streptomyces* sp. VITGV156 grown on ISP2 medium for 15 days (Veilumuthu P. and Christopher J. G., 2022). The DNA libraries, primed for sequencing, were adeptly prepared utilizing the TruSeq Nano kit. The subsequent sequencing procedure was conducted using the Illumina NextSeq 500 platform, under the stewardship of Macrogen Eurofins Genomics India Pt. Ltd. This approach generated paired-end reads with a length of 150 bp, ensuring that the vast majority—more than 20—exhibited Phred scores of Q30 or higher. After the read acquisition, a trimming process was executed using a sliding window of 10 bp, employing a threshold of 20. The processed reads were then orchestrated into cohesive scaffolds via the SPAdes assembler (v-3.13.0). These scaffolds were judiciously juxtaposed with homologous sequences to discern the most closely related organism(s). The genome of VITGV156, annotated using Prokka (version 1.12). Annotation of protein-coding and RNA genes was performed using the final assembled draft genome. Gene prediction was conducted with Prokka (version 1.12), while pathway annotation was completed by aligning predicted genes against the curated KEGG GENES database through the KAAS (KEGG Automatic Annotation Server). The genome, designated as *Streptomyces* ferrovit, is available on NCBI under BioProject ID PRJNA750872 (Grant and Stothard, 2008). A circular representation of the genome was generated using the CGView server. Phylogenetic analysis, based on 16S ribosomal RNA sequences retrieved from NCBI, was performed with MegaX software (Kumar et al., 2018). Biosynthetic gene clusters responsible for secondary metabolite production were identified using antiSMASH 6.0 (Blin et al., 2021) and BAGEL 4 tools (van Heel et al., 2018).

### 2.5 Gene Ontology analysis

The genome of *Streptomyces* sp. VITGV156 was annotated using Blast2GO platform, using the Gene Ontology (GO) framework. This bioinformatics tool seamlessly facilitated the assignment of succinct GO annotations to our gene sequences, illuminating their biological roles and properties within a meticulously constructed hierarchical vocabulary. The GO structure is thoughtfully compartmentalized into three cardinal categories, namely Cellular Component (CC), Molecular Function (MF), and Biological Process (BP), each intricately outlining the functional context of the gene products. In the mechanics of this annotation process, the platform nimbly navigates through two indispensable NCBI mapping files, namely, gene_info and gene2accession. By ingeniously correlating Blast accessions with gene names or symbols, it strategically ascertains the relevant GO terms from the expansive GO database. Furthermore, a visual representation of the distribution of these GO terms is ingeniously orchestrated through the WEGO (Web Gene Ontology Annotation plot) portal, thereby enhancing the interpretive dimension of our findings and insights (http://wego.genomics.org.cn/cgi-bin/wego/index.pl) (Veilumuthu et al., 2022).

### 2.6 Comparative genome analysis

*Streptomyces* sp. VITGV156 the degree of relatedness between these two strains was judiciously quantified via the Average Nucleotide Identity (ANI) and digital DNA-DNA Hybridization (dDDH) metrics, by the method elucidated by Meier-Kolthoff et al. (2014). To this end, the Ortho ANI v0.93.1 algorithm embedded within EzBioCloud was adroitly harnessed to gauge the overarching similarity of orthologous genome fragments, thereby encapsulating the essence of their relationship (Lee and Whang, 2016).

### 2.7 Molecular docking analysis

#### 2.7.1 Protein preparation

The 3D crystal structure of PBP2a (Penicillin Binding Protein 2a) from Methicillin Resistant *Staphylococcus aureus* (MRSA) (PDB ID: 5M18-Chain A, Resolution: 1.98 Å) and the crystal structure of TLA-1 extended spectrum beta-lactamase from *E. coli* (PDB ID: 6NVU- Chain A, Resolution: 2.50 Å) were retrieved from Protein Data Bank (PDB) database in PDB format. Prior to docking, the protein structures were prepared using AutoDock Vina software, involving the removal of heteroatoms and water molecules, as well as the addition of polar hydrogen atoms and Kollman charges. The proteins were then saved in pdbqt format (Deepasree and Subhashree, 2023). This is done to purify the target protein and remove undesirable molecules and separate protein molecules from the built-in ligand. With each protein’s corresponding inhibitory ligand affixed to its core, a grid box was constructed (Pisano et al., 2019). Grid parameters were set for PDB ID: 5M18 (5.36, −13.274, −45.357) for (x, y, z) and PDB ID: 6NVU (8.375, 21.71, 15.526) for (x, y, z). The config. txt file was used to calculate the grid box measurements for AutoDock Vina docking (Bhandari et al., 2022).

#### 2.7.2 Preparation of ligand

Forty-five compounds identified from *Streptomyces* sp. VITGV156 via GC-MS analysis were prepared for docking. SMILES data for each compound were retrieved from the PubChem database and converted into 3D PDB structures using the NCI/CADD online converter. Metabolite preparation involved adding Gasteiger charges, assigning aromatic carbons, detecting roots, and saving in pdbqt format through AutoDock Vina (Aragón-Muriel et al., 2021). Drug-likeness properties were evaluated using SwissADME based on Lipinski’s rule of five, which assesses bond donors, bond acceptors, logP, and molecular weight criteria (Naqvi et al., 2019).

#### 2.7.3 Molecular docking and visualization

The software AutoDock Vina was used to do the molecular docking. Of all the docking algorithms, AutoDock Vina proved to be the most effective at executing blind docking and producing postures that optimally bind deep within the 5 Å of the binding pocket (Azam and Abbasi, 2013). Grid box attributes from the configuration file and protein and ligand information were used by the software for docking. It uses a global optimizer with repeated local search. Docking results were studied in terms of binding affinity binding energy or binding score. A ligand’s binding energy indicates its ability to completely attach to the active site of the target receptor (Yuan et al., 2017). The metabolite exhibiting the lowest binding energy is considered to be the best active compound because stronger bonds are established when affinity energy is lower, and *vice versa*. Three such active compounds for each protein were selected for subsequent visualization (Shah et al., 2023).

The selected docked complexes were visualized using Ligplot+ and PyMOL software (Rifda et al., 2023). With the aid of the program Ligplot+, the interaction of ligand and amino acid residues of the protein involved in the binding process was examined. We looked at the two primary types of intermolecular interactions: hydrophobic and hydrogen bond interactions. Additionally, Pymol software was employed to visualize the protein-ligand combination in three dimensions (Deepasree and Subhashree, 2023; Al-Noor et al., 2021).

#### 2.7.4 Toxicity analysis

The OSIRIS instrument was employed to conduct a toxicological analysis. Based on ADMET, OSIRIS is a comprehensive internal drug discovery informatics system that consists of a Java library layer with reusable cheminformatics capabilities. Using *in silico*, it was used to predict the total drug score. Characteristics like mutagenicity, tumorigenicity, irritability, or reproductive consequences that are associated with an increased risk are designated as “red”, whereas behavior that complies with pharmacological regulations is indicated as “green” (Vijesh et al., 2013).

#### 2.7.5 Biological activity analysis

To examine the biological activity of the discovered secondary metabolites, the PASS online tool was used. Because this prediction is based solely on the chemical structural formula, it is possible for virtual structures that have been built in a computer but have not yet been synthesized. Pa and Pi, which have values between 0 and 1, represent possible in-activity and activity, respectively. The highest level of activity is represented by a one, and the lowest level by a zero. On the pass online result scale, antimicrobial activity was graded from 0 (inactive) to 1 (active). When the Pa score is greater than 0.7, it signifies exceptionally strong antimicrobial activity, >0.5, good antimicrobial activity, and >0.3, low antimicrobial activity (Tiwari et al., 2021).

#### 2.7.6 Data availability

The whole genome of *Streptomyces* sp. VITGV156 was submitted to the NCBI SRA portal under BioProject accession number PRJNA750872, BioSample accession number SAMN20499087, SRA accession number SRS9645416 and Accession ID: 20499087; NCMR (Culture Deposit Accession Number – MCC4965).

## 3 Results

### 3.1 Selection and primary screening of *Streptomyces* sp. VITGV156

**FIGURE 1:**
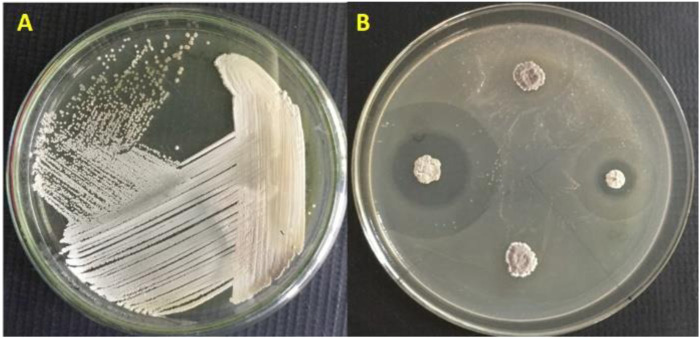
**(A)** Colony morphology of *Streptomyces sp.* VITGV156, **(B)** Antibacterial screening revealed that VITGV156 exhibited inhibition *E. coli*.

The *Streptomyces* sp. VITGV156 cultured on on ISP2 agar plates is given in Figure 1A. A comprehensive insight, Figure 1A encapsulates its distinctive cultural attributes and colony morphology. Individual colonies were selected and purified by streaking. A broad range of compounds produced by *Streptomyces* sp. were reported to exhibit antimicrobial activity. Therefore, a preliminary screening was done by assessing the antimicrobial activity of the isolated strains by visualizing their activity in *E. coli*. One of the isolates exhibited significant activity by exhibiting a large zone of clearance (Figure 1B). The strain was named *Streptomyces* sp. VITGV156 and characterized further. Phenotypic characterization was done to support the novelist of VITGV156. Up on growth, they form aerial mycelium on the surface. The color of substrate mycelium is pale yellow on ISP2.

### 3.2 Crude extract of *Streptomyces* sp. VITGV156 exhibited antibacterial properties

**FIGURE 2:**
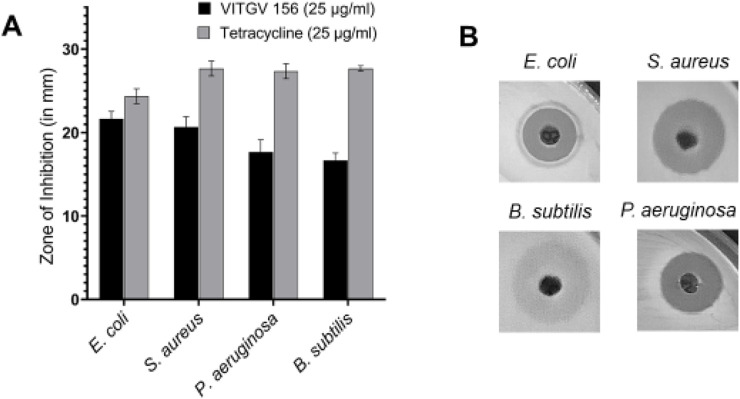
Antibacterial activity of VITGV 156 against pathogenic bacterial strains. **(A)** The zone of inhibition (in mm) of VITGV 156 (25 μg/mL) compared to tetracycline (25 μg/mL) against *E. coli, S. aureus, P. aeruginosa,* and *B. subtilis*. The results are presented as mean ± standard deviation (n = 3). **(B)** Representative images of agar diffusion assay showing the inhibition zones for *E. coli*, *S. aureus, P. aeruginosa,* and *B. subtilis* treated with VITGV 156.

To extract the secondary metabolites ethyl acetate was used from the culture supernatant of *Streptomyces* sp. VITGV156. The antimicrobial activity of *Streptomyces* sp. VITGV156 was assessed using the diffusion technique at using various concentrations. Antimicrobial activity as evaluated against 4 pathogenic bacterial strains (*E. coli, S. aureus, P. aeruginosa,* and *B. subtilis*). Tetracycline (25 μg/mL) was used as a positive control. The ethyl acetate extract of *Streptomyces* sp. VITGV156 exhibited antimicrobial activity against all tested organisms*,* (Figures 2A, B **)**, which is observed from the zone of inhibition. Ethyl acetate extract exhibited strong antibacterial activity against *E. coli* (21.6 mm) followed by *S. aureus* (20.6 mm), *P. aeruginosa* (17.6 mm), and *B. subtilis* (16.6 mm).

### 3.3 GC-MS analysis

**FIGURE 3:**
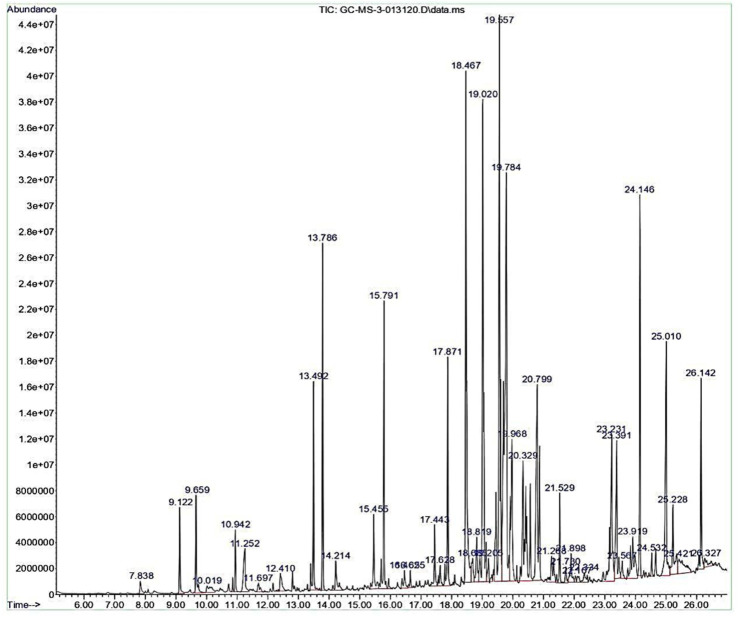
GC chromatogram representing the separation of volatile compounds in the ethyl acetate crude extract of *Streptomyces* sp. VITGV156. Peaks correspond to different chemical constituents identified based on retention time.

The crude extract was analysed in GC-MS to ascertain the array of secondary metabolites. The GC-MS chromatogram of the ethyl acetate extract of *Streptomyces* sp. VITGV156 exhibited 45 distinct peaks (Figure 3). Details of the compounds, including their retention times, molecular formulas, molecular weights, and concentrations (expressed as peak area percentages), are provided in Supplementary Table S1. Among the secondary metabolites, compounds like Di-sec-butyl phthalate (10.94%), N-Acetyltyramine (12.20%), Dibutyl phthalate (8.19%), 1,2-Benzenedicarboxylic acid, bis(1-methylethyl) ester (7.71%) were identified in high concentration. The rest compounds were present in lower quantities with (0.24 t0 5.49%). Interestingly, commercially important compound squalene was observed at a retention time of 25.42 min with the peak area coverage of 1.27% (Supplementary Figure S1).

**FIGURE 4:**
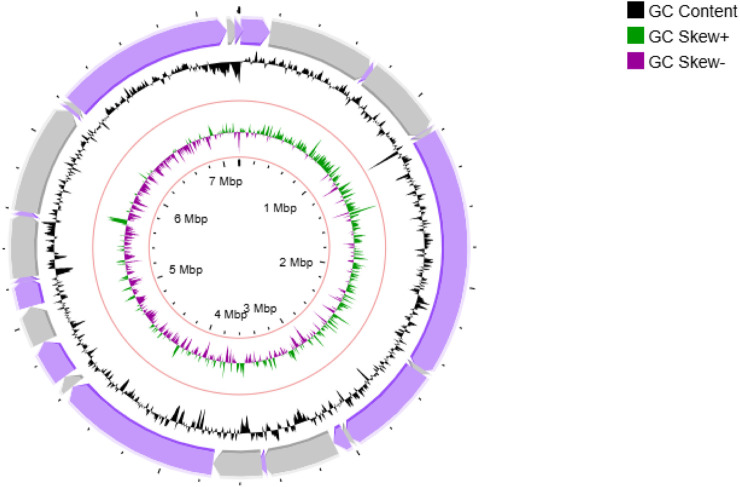
Circular genome map of *Streptomyces* sp. VITGV156. The following features are shown (moving from the outermost track to the innermost track); origin of replication positioned: ORF genes in violet color, positive and negative GC content skew in green and purple respectively. GC content (black) and the genome position in the center. The innermost ring displays the overall genome size of *Streptomyces* sp. VITGV156.

### 3.4 Genome analysis

Sequencing of Streptomyces sp. VITGV156 revealed a linear genome spanning 7,207,566 base pairs with a G+C content of 72.61% (Figure 4). Assembly of the genome resulted in 201 scaffolds with an N50 value of 437,096 base pairs. The annotated genome comprises 6,259 protein-coding genes, along with 5 rRNA and 81 tRNA operons (Table 1).

**TABLE 1:** Genomic features of strain *Streptomyces* sp. VITGV156.

Feature	Value
Genome topology	linear
Total genome size (bp)	8.18 Mb
Assembly size (bp)	7,207,566 bp
G + C content (%)	72.61
Average Nucleotide Identity (ANI)^a^ (%)	91.22
Digital DNA-DNA Hybridization (dDDH)^a^ (%)	44.00
Coding DNA sequence	6,386
Protein coding genes	6,259
Unique genes (of unknown function)	151
Total no. of rRNA operons	5
Total no. of tRNA operons	81
BioSample ID	20499087

^a^ Compared with *Streptomyces mutabilis* strain TRM45540.

It is notable that *Streptomyces* sp. VITGV156 falls on a close clade with *S. luteus* TRM45540 and this strain was used as a reference strain in genome analysis. The Average Nucleotide Identity value dawned at 91.2%, accompanied by a dDDH value of 44.0% (Table 1). All these unveil, *Streptomyces* sp. VITGV156 as a novel hitherto unreported strain. The strain was submitted to National Centre for Microbial Resource (NCMR) (accession no: MCC4965). The comprehensive analysis of the Gene Ontology of VITGV156 revealed that out of the total coding genes, a total of 1,781 genes harmonizes their roles in catalytic activities, bindings, and transportation, whereas 1,315 genes are involved in other biological functions and 851 contribute to the structural components for the cell.

The whole genome sequence of *Streptomyces* sp. VITGV156 was analyzed using antiSMASH tools to identify biosynthetic gene clusters (BGCs) associated with secondary metabolite production. The analysis revealed twenty nine putative BGCs, including clusters encoding non-ribosomal peptide synthetases (NRPS), ribosomally synthesized and post-translationally modified peptides (RiPPs), and siderophore compounds. Among these, three clusters showed less than 20% similarity to known natural products, indicating the presence of potentially novel compounds. Of the 29 identified BGCs, 34% (10 clusters) were associated with the biosynthesis of peptide compounds, 24% (7 clusters) with polyketide production, 13% (4 clusters) with terpene synthesis, and 10% (3 clusters) with siderophore formation. Additionally, the strain produced an indole-based compound structurally distinct from 5-isoprenylindole-3-carboxylate β-D-glycosyl ester, suggesting it may represent an unidentified metabolite. This genomic insight highlights the potential of *Streptomyces sp.* VITGV156 as a source of diverse and novel bioactive secondary metabolites (Supplementary Table S2).

### 3.5 ADME analysis

Every molecule must be evaluated for drug-likeness at the outset of drug development. Each of the 45 compounds was examined for its potential to be a medication, as per the GC-MS report (Supplementary Table S3). All of the ligands in this case were determined to have molecular weights between 98 and 536 g/mol. The lowest molecular weight found for 1H-Pyrazole, 4,5-dihydro-5,5-dimethyl-4-isopropylidene (ligand 2) was 98.14 g/mol. The greatest molecular weight of ligand 26, 2,3-Dimethyl-1-hexene, was 536 g/mol. Out of 45 compounds, 17 have one violation, which can be justified by a high log P value (>6), whereas the remaining compounds have no violations. This finding suggests that oral consumption of all 45 chemicals is possible (Supplementary Table S3).

### 3.6 Docking

Among the *in silico* techniques used in drug discovery, molecular docking serves three primary functions: finding new ligands; estimating the bond structure of active ligands; and forecasting the conformation and affinity of these ligands. This approach is among the more basic and straightforward ones. Supplementary Table S4 displayed the outcomes of the molecular docking investigation. All 45 secondary metabolites’ affinities in terms of binding energy against two target protein receptors, such as 5M18 and 6NVU, were predicted by the *in silico* molecular docking experiments. The range of binding affinities against 5M18 and 6NVU were determined to be, respectively, −4.0 to −7.5 kcal/mol and −3.9 to −7.2 kcal/mol. The best candidates were selected based on high negative binding affinity values, which show a compound’s higher ability to block the virulence protein (Table 2). The ligands Benzo [h]quinoline, 2,4-dimethyl- (ligand 17), Squalene (ligand 43), and Fumaric acid, monoamide, N-benzyl-N-phenylethyl-, ethyl ester (ligand 38) have binding affinities of −7.5, −7.3, and −7.2 kcal/mol, respectively. The 5M18 protein receptor had the highest binding affinity for these drugs. For the 6NVU protein receptor, the binding affinities of ligands Benzo [h]quinoline, 2,4-dimethyl- (ligand 17), 2,5-Piperazinedione, 3-(hydroxymethyl)-6-(phenylmethyl)- (ligand 40), and Fumaric acid, monoamide, N-benzyl-N-phenylethyl-, ethyl ester (ligand 38) are, respectively, −7.2, −6.8, and −6.6 kcal/mol. The docked metabolite complexes were then visualized, and the amino acid interaction was examined using Pymol and Ligplot+ and maestro from Schrödinger packages.

**TABLE 2:** Binding affinity extracted compounds from *Streptomyces* sp. VITGV156.

S. No	Chemical compound	Binding energy (Kcal/mol)	
		PBP2a	Beta-lactamase
1	Benzo [h]quinoline, 2,4-dimethyl-	−7.5	−7.2
2	Fumaric acid, monoamide, N-benzyl-N-phenylethyl-, ethyl ester	−7.2	−6.6
3	2,5-Piperazinedione, 3-(hydroxymethyl)-6-(phenylmethyl)-	−6.9	−6.8
4	Squalene	−7.3	−4.8

**FIGURE 5:**
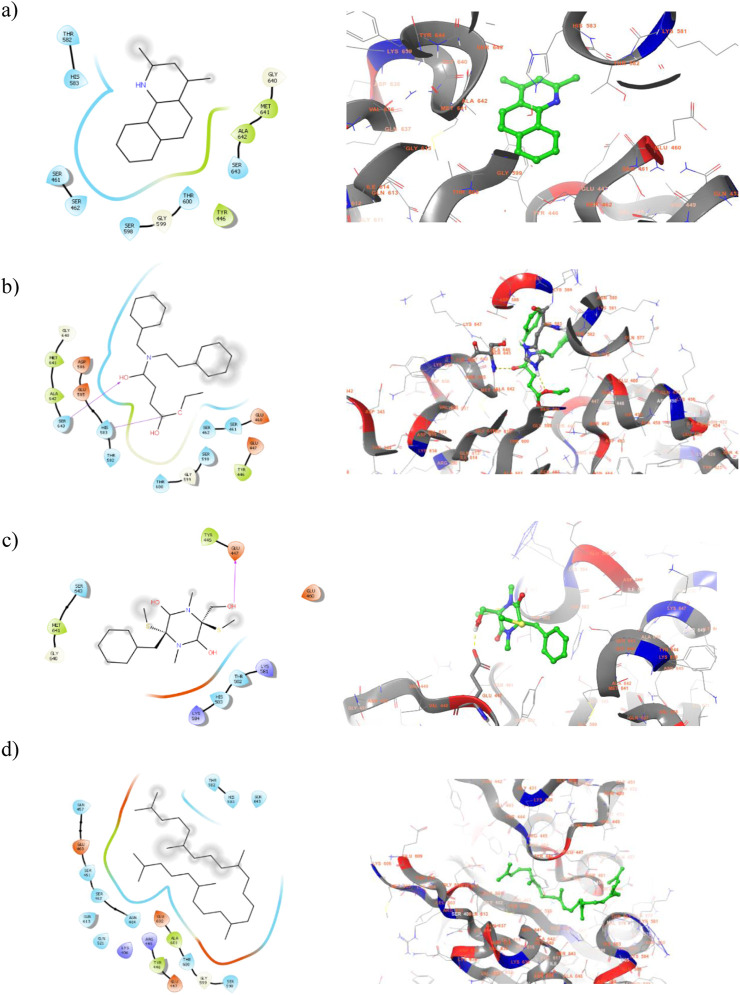
2D (Left) and 3D (Right) visualization of active secondary metabolites with the protein PBP2a (PDB ID: 5m18) **(a)** ligand 17-; **(b)** ligand 38 **(c)** ligand 40 **(d)** ligand 43.

**FIGURE 6:**
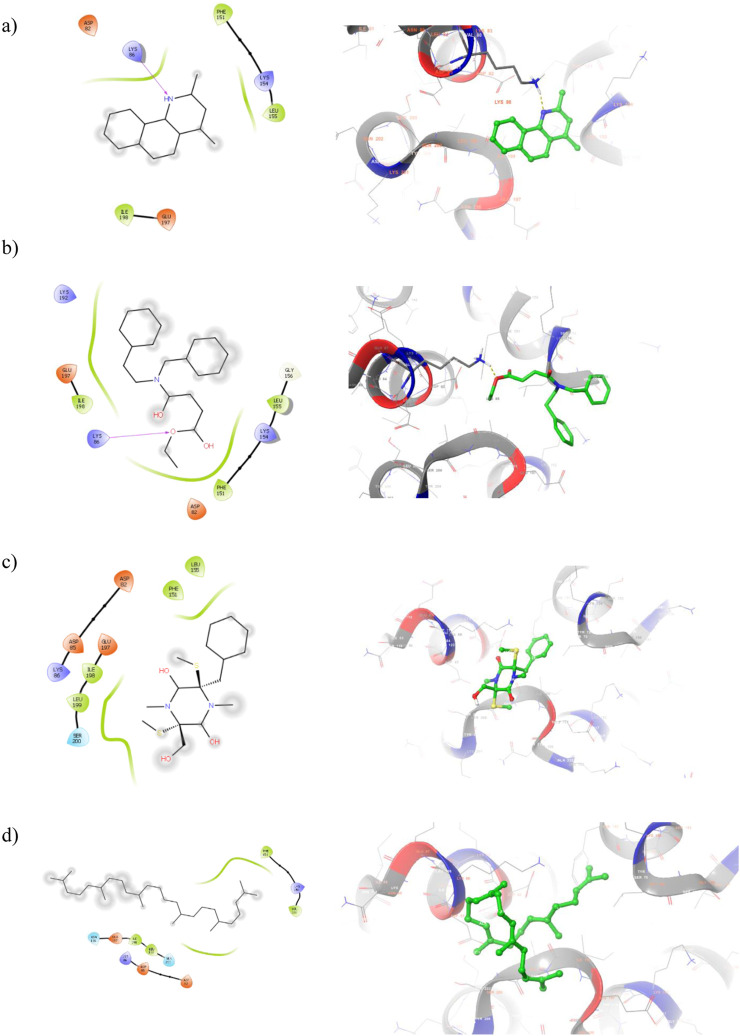
2D (Left) and 3D (Right) visualization of active secondary metabolites with the protein Beta-lactamase (PDB ID: 6VNU) **(a)** ligand 17-; **(b)** ligand 38 **(c)** ligand 40 **(d)** ligand 43.

2D and 3D visualization of the top ranked compounds with PBP2a protein is represented in Figure 5. It is to note that the compound ligand 38 and ligand 40 displayed two and one hydrogen bond interaction with PBP2 binding site residues. For instance, SER 643 and HIS 583 are formed hydrogen bond interaction with ligand 38. Similarly, alcohol group of ligand 40 interact with GLU 447 of target protein. Although hydrogen bond interactions were not observed in ligand 17 and ligand 43, hydrophobic interactions were majorly contribute to the compound binding process. The hydrophobic interaction between ligand 17 and TYR 446, MET 641, and ALA 642 helps to anchor the ligand into binding site residues. These interactions suggest strong binding affinity, potentially interfering with bacterial cell wall synthesis. Moreover, this is the sign of competitive inhibition which mimic binding of β-lactam antibiotic. Molecular interactions of the compound with Beta-lactamase protein presented in Figure 6. One hydrogen bond interaction was observed between ligand 17 and LYS 86. Similar interacting pattern was observed in the case of ligand 38 and LYS 86 residue. Other compound interactions were majorly contributed by hydrophobic interactions.

### 3.7 Toxicity analysis

To assess the possible harmful impact of the extract on mammalian cells, a toxicological prediction was made. With the use of OSIRIS, a free programme that forecasts molecular characteristics related to physico-chemistry and toxicity, we may create pharmaceutically active molecules with the best possible features. All 45 compounds were submitted for OSIRIS toxicity testing (Supplementary Table S5). Among them, the above selected compounds Benzo [h]quinoline, 2,4-dimethyl- (ligand 17), 2,5-Piperazinedione, 3-(hydroxymethyl)-6-(phenylmethyl)- (ligand 40), and Squalene (ligand 43) shows less toxicity risks in all categories, while Fumaric acid, monoamide, N-benzyl-N-phenylethyl-, ethyl ester (ligand 38) shows less toxicity towards mutagenic, tumorigenic, and reproductive effective but high toxicity towards irritation. In addition to that all the selected compounds exhibit poor drug-likeness activity except 2,5-Piperazinedione, 3-(hydroxymethyl)-6-(phenylmethyl)- (ligand 40) (Table 3).

**TABLE 3:** Toxicity analysis of extracted compounds from *Streptomyces* sp. VITGV156.

S. No	Chemical compound	Mutagenic	Tumorigenic	Irritant	Reproductive effective	Drug likeness
1	Benzo [h]quinoline, 2,4-dimethyl-	Yellow	Yellow	Green	Green	−3.84
2	Fumaric acid, monoamide, N-benzyl-N-phenylethyl-, ethyl ester	Green	Green	Red	Green	−6.39
3	2,5-Piperazinedione, 3-(hydroxymethyl)-6-(phenylmethyl)-	Green	Green	Green	Green	6.12
4	Squalene	Green	Green	Green	Green	−3.52

### 3.8 Biological activity

The PASS online server was used to assess the biological activity of the secondary metabolites from VITGV38 that had been found. Biological activities such as antibiotic, antifungal, and antibacterial qualities are tested. In terms of Pa and Pi, the biological activity is supplied by the server. When it comes to biological activity (antibacterial, antifungal, and antibiotic), a Pa score of >0.7 denotes outstanding activity, 0.5 good activity, and <0.3 poor activity. On the other side, the Pi value, which ranges from 0 to 1, reflects potential activity and inactivity, respectively. The average Pa score for all the compounds ranges from 0.014 (low activity) to 0.595 (high activity). The compound Benzo [h]quinoline, 2,4-dimethyl- (ligand 17) shows poor antibacterial and antifungal activity (Table 4). The compound Fumaric acid, monoamide, N-benzyl-N-phenylethyl-, and ethyl ester (ligand 38) shows poor antifungal activity. Squalene (ligand 43) has good antibacterial and antifungal activity. The compound 2,5-Piperazinedione, 3-(hydroxymethyl)-6-(phenylmethyl)- (ligand 40) exhibits good antifungal activity (Supplementary Table S6).

**TABLE 4:** Biological activity of extracted compounds from *Streptomyces* sp. VITGV156.

S. No	Chemical compound	Antibacterial activity (Pa-value)	Antifungal activity (Pa- value)	Antibiotic activity (Pa-value)
1	Benzo [h]quinoline, 2,4-dimethyl-	0.137	0.203	-
2	Fumaric acid, monoamide, N-benzyl-N-phenylethyl-, ethyl ester	-	0.237	-
3	2,5-Piperazinedione, 3-(hydroxymethyl)-6-(phenylmethyl)-	0.293	0.436	0.155
4	Squalene	0.397	0.531	0.194

## 4 Discussion

This study explores the genomic potential of *Streptomyces sp.* VITGV156, emphasizing its ability to produce secondary metabolites with antimicrobial properties. The strain demonstrated significant antibacterial activity, underscoring its capacity to synthesize bioactive compounds. The morphological features of *Streptomyces sp.* VITGV156, including its colony structure and spore formation, align with the characteristic traits of the *Streptomyces* genus. These findings are consistent with observations from other studies, reinforcing its classification and potential as a source of novel antimicrobials (Aouar et al., 2012; Bhandari et al., 2022). The identification of secondary metabolite biosynthetic gene clusters further validates the strain’s capability to produce a variety of antimicrobial compounds. Comparative studies have shown that strains of *Streptomyces* are prolific producers of diverse bioactive metabolites, often responsible for their observed antibacterial efficacy. The alignment of VITGV156s genomic and phenotypic attributes with those documented in literature highlights its significance in the search for new antibiotics. This study aims to reveals the genomic potent of *Streptomyces* sp. VITGV156 and its potential to produce secondary metabolites of antimicrobial value. A strain *Streptomyces* sp. VITGV156 exhibited significant antibacterial activity which exhibited their secondary metabolite potential. Preliminary analysis of the ethyl acetate extract of the VITGV156 exhibited antimicrobial activity (Kawahara et al., 2018; Gomez-Escribano et al., 2019; Nguyen et al., 2020). However, the VITGV156 colony forms a clear zone of inhibition when grown with *E. coli* (Figure 1B). The limited antimicrobial activity could be enhanced upon optimization of fermentation conditions. The GC-MS analysis of the ethyl acetate extract revealed the presence of various volatile compounds, many of which are commercially valuable. Similar compounds have been previously reported in studies on *Streptomyces* metabolites, highlighting their significance in bioactive molecule production (El-Naggar et al., 2017; Usha Nandhini et al., 2018; Al-Dhabi et al., 2020; Tangjitjaroenkun et al., 2021). Notable compounds identified in the extract, such as Benzo [h]quinoline, 2,4-dimethyl-; Fumaric acid monoamide, N-benzyl-N-phenylethyl-, ethyl ester; 2,5-Piperazinedione, 3-(hydroxymethyl)-6-(phenylmethyl)-; and Squalene, are potentially responsible for the observed antimicrobial activity.

Optimization of fermentation condition potentially impact secondary metabolite production. Many factors such as pH, temperature, carbon source, nitrogen source and minerals are affecting the production of secondary metabolites. Barka et al. (2016) has resulted that the production of secondary metabolites of *Streptomyces* sp. has improved by adjusting the carbon and nitrogen source ratios. Similarly, controlled pH regulation helps to increase the production of polyketides and nonribosomal peptides from *Streptomyces* sp. (Hodgson, 2000). These strategies provide a foundation for future investigations to enhance the yield and bioactivity of secondary metabolites from *Streptomyces* sp. VITGV156.

Whole-genome sequencing provided detailed insights into the genomic composition of *Streptomyces sp.* VITGV156, revealing 29 biosynthetic gene clusters (BGCs). Of these, 26 showed significant similarity to known BGCs, indicating their potential role in secondary metabolite production. Antimicrobial compounds such as nystatin, fluostatins, coelichelin, vicenistatin, informatipeptin, versipelostatin, butyrolactone, herboxidiene, paulomycin, methylenomycin A, and sipanmycin were identified, showcasing the strain’s capacity for generating bioactive molecules. This genomic richness highlights *S. sp.* VITGV156 as a promising source for novel antimicrobial derivatives and other bioactive compounds, reinforcing its importance in the development of new therapeutic agents. These compounds have been previously documented in various *Streptomyces* strains: coelichelin in *Streptomyces coelicolor* [23], fluostatins in *Streptomyces* strain Acta 1383 [24], vicenistatin in *Streptomyces halstedii* [25], nystatin in *Streptomyces noursei* strain BSM1 [26], sipanmycin in *Streptomyces hygroscopicus* MTCC 4003 [27], and informatipeptin in *Streptomyces viridochromogenes* DSM 40736 [28]. The genome of *Streptomyces sp.* VITGV156 reveals an intriguing mix of gene clusters, including those with 100% similarity, some with no resemblance, and others showing minimal similarity (less than 10%), which highlights the strain’s potential to produce a diverse array of bioactive secondary metabolites. This variability in the gene clusters adds to the allure of VITGV156 as a promising producer of novel compounds.

The genome of VITGV156 harbors genes that are essential for the synthesis of various antimicrobial compounds, such as non-ribosomal peptide synthetases (NRPS) and Ribosomally Synthesized and Post-translationally Modified Peptides (RiPPs). NRPS enzymes are responsible for the biosynthesis of nonribosomal peptides, which have broad biological applications, including antimicrobial activity. Some of these nonribosomal peptides serve as last-resort antibiotics due to their potent effects against resistant pathogens (Walsh, 2008). VITGV156 encodes at least five distinct NRPS, one of which is known to produce the antibacterial compound Streptothricin (Supplementary Table S2) (Morgan et al., 2023). However, the full potential of the other NRPS products remains unidentified. RiPPs are another important class of antimicrobial peptides, synthesized on ribosomes and later modified post-translationally. Due to their wide antibacterial spectrum against both gram-positive and gram-negative bacteria, RiPPs have become essential in the food and pharmaceutical industries (Van Kraaij et al., 1999; Arnison et al., 2013).

The VITGV156 genome codes for four RiPPs, two of which show limited similarity to known compounds, suggesting the possibility of novel peptides with untapped antimicrobial potential (Supplementary Table S2). These findings align with previous research on other *Streptomyces* species. For example, *Streptomyces sp.* Babs14 possesses 29 biosynthetic gene clusters, with eight showing full alignment with known clusters (Zerouki et al., 2021). Similarly, *Streptomyces sp.* CC77 was found to produce siderophores, a class of secondary metabolites with crucial roles in iron acquisition, further expanding our understanding of the metabolic diversity in *Streptomyces* (Cruz-Morales et al., 2017). Moreover, *Streptomyces sp.* ICC1 has been shown to harbor 37 biosynthetic gene clusters, indicating the vast biosynthetic potential within the genus (Gosse et al., 2019). Additionally, the VITGV156 genome contains genes involved in the production of other valuable metabolites, such as butyrolactone, siderophores, indole, ectoine, and terpenes. Notably, the strain produces squalene, a compound detected in its culture extract, further supporting the potential of *Streptomyces sp.* VITGV156 as a source of novel bioactive compounds with significant pharmaceutical and industrial applications (Supplementary Figure S1).

Squalene, a naturally occurring triterpene hydrocarbon molecule (C_30_H50), occupies a pivotal position due to its diverse applications beyond its role as a precursor to myriad bioactive compounds including steroids and hopanoids (Wołosik et al., 2013). Initially purified from shark liver oil, microbial production of squalene has been proven promising due to the feasibility of industrial fermentation availabilities. Squalene production from microbial sources is via two isoprenoid pathways which involve enzymatic conversion of acetyl-CoA to squalene (Ghimire et al., 2009; Katabami et al., 2015; Gohil et al., 2019).

Blast analysis of the VITGV156 genome revealed the presence of genes encoding squalene diphosphate synthase (*hpnD*) and squalene synthase (*hpnC*), responsible for squalene synthesis. The presence of these enzymes further validates the involvement of the MVA pathway in squalene biosynthesis in VITGV156. The presence of squalene in the culture medium implies that it is not concealed within cryptic genes. Enhancement in squalene production to 1.27% can potentially be achieved through the identification of suitable elicitors or promoters. Squalene, classified as a pentacyclic triterpene, is synthesized by *Streptomyces* sp. through the methylerythritol phosphate pathway (Fontana et al., 2001). These results serve as empirical evidence that *Streptomyces* sp. VITGV156 is genuinely prolific in generating an array of bioactive secondary metabolites.

A molecular docking investigation was conducted to identify the binding site to limit the function of MRSA protein PBP2a (Penicillin-binding protein) (PDB ID:5m18) and Beta-lactamase (PDB ID:6nvu). These targets engage themselves in antibiotic resistance mechanisms especially against Beta-lactam antibiotics. PBP2a is a membrane-bound protein that plays a major role in antibiotic resistance seen in MRSA. It is a transpeptidase with reduced affinity for beta-lactam antibiotics. This allows the MRSA strain to synthesize cell walls even in the presence of antibiotics (Dordel et al., 2014). Beta-lactamase is an enzyme produced by certain bacteria that confers resistance to beta-lactam antibiotics (Ghafourian et al., 2015). This investigation showed that the active secondary metabolites derived from the *Streptomyces* sp. VITGV156 can inhibit these targets. Among the metabolites Squalene (ligand 40) and Benzo [h]quinoline, 2,4-dimethyl- (ligand 17) has the highest binding affinity towards these targeted receptors. Further molecular dynamics simulations and experimental validation of these compounds could offer clinicians better insight into their potential as therapeutic agents (Saeed M. et al., 2024; Saeed A. et al., 2024).

The candidate molecule squalene and benzo [h]quinoline, 2,4-dimethyl-, demonstrated significant anti-microbial activity against resistant proteins. Given these properties the compounds might become potential antibiotics against multi-drug resistance pathogens such as MSRA. It is important to note that squalene is generally known for its potential antimicrobial and antifungal properties indicating its broader pharmaceutical benefits. The presence of biosynthetic gene clusters (BGCs) encoding nonribosomal peptide synthetases (NRPS) and ribosomally synthesized and post-translationally modified peptides (RiPPs) indicates the strain’s capacity for metabolic engineering and biotechnological optimization to enhance metabolite yield. The production of this potential metabolites via fermentation of *Streptomyces sp*. VITGV156 provides an alternative, sustainable microbial source, which could reduce dependency on traditional sources like shark liver oil, aligning with the growing demand for eco-friendly bioproduction methods in the pharmaceutical and cosmetics industries. Formulation studies and *in vivo* validation of these compounds will be the next key steps in advancing them toward pharmaceutical applications.

## 5 Conclusion

In the present study pinpoint the genomic and metabolic ability of *Streptomyces* sp. VITGV156 as a potential source of secondary metabolites with promising anti-microbial properties. Our study results reveal that the strain capable of producing significant quantity of secondary metabolites and therapeutic compounds. Whole-genome sequencing revealed 29 biosynthetic gene clusters, including those responsible for the synthesis of antibiotics such as nystatin, fluostatins, coelichelin, and sipanmycin. Additionally, the identification of Nonribosomal Peptide Synthetases (NRPS) and Ribosomally Synthesized and Post-translationally Modified Peptides (RiPPs) further underscores the strain’s potential in producing novel antimicrobial agents. GC-MS analysis reveal that the ethyl acetate exact of VITGV156 having commercially important volatile compounds including squalene. The detection of genes encoding key enzymes involved in squalene biosynthesis, such as hpnD and hpnC, suggests a metabolic framework conducive to the production of bioactive terpenoids. Molecular docking studies reveal that two compounds such as squalene and Benzo [h]quinoline, 2,4-dimethyl- were possess higher binding capability against PBP2a and Beta-lactamase, bacterial resistance proteins. While these results establish the strain’s promising antimicrobial capabilities, further experimental validation, including optimization of fermentation conditions and *in vivo* studies, is necessary to fully explore its potential in drug development. The genomic diversity and metabolic versatility of *Streptomyces* sp. VITGV156 position it as an invaluable resource for discovering novel antimicrobial agents, contributing to the ongoing fight against antibiotic resistance. These computational approaches facilitate the prediction of novel bioactive compounds, advancing the field of bioinformatics and its role in enhancing our understanding of microbial genomics. This research not only contributes to the growing repository of microbial genomes but also opens up new possibilities for leveraging endophytic microbes in developing novel antimicrobial agents, ultimately addressing global challenges in antimicrobial resistance and public health.

## Data Availability

The datasets presented in this study can be found in online repositories. The names of the repository/repositories and accession number(s) can be found below: https://www.ncbi.nlm.nih.gov/biosample/20499087.
