# Supplementary material for: Streptomyces sp. VITGV156 secondary metabolite binds pathogenic protein PBP2a and Beta-lactamase
Source: Front Bioinform. 2025 Mar 26;5:1544800. doi: 10.3389/fbinf.2025.1544800 (PMC11979255; doi:10.3389/fbinf.2025.1544800)
Supplement: Supplementary file 1 [file Table1.docx]

*Streptomyces* sp. VITGV156 secondary metabolite binds pathogenic protein PBP2a and Beta-lactamase

Veilumuthu Pattapulavar^1^, Sathiyabama Ramanujam^2^, Manisha Shah^3^, Muthu Kumar Thirunavukkarasu^4^, Sivakumar Arumugam^3^, Ramanathan Karuppasamy^5^, Antony V. Samrot^6^, K. Deepasree^7^, Subhashree Venugopal^7^ and John Godwin Christopher^1^*

^1^Department of Biomedical Sciences, School of BioSciences and Technology, Vellore Institute of Technology, Vellore, India.

^2^Department of Science and Humanities, Karpagam Academy of Higher Education, Coimbatore, Tamil Nadu, India- 641021.

^3^Department of Bio-Sciences, School of BioSciences and Technology, Vellore Institute of Technology, Vellore, India, ^4^School of Sciences and Humanities, SR University, Warangal, Telangana – 506371, India

^5^Department of Biotechnology, School of BioSciences and Technology, Vellore Institute of Technology, Vellore, India,

^6^Department of Microbiology, Faculty of Medicine, Manipal University College Malaysia, Melaka 75150.

^7^Department of Integrative Biology, School of BioSciences and Technology, Vellore Institute of Technology, Vellore, India

**Supporting information**

**Table S1. GC-MS chromatogram of the crude extract of Streptomyces sp. VITGV156**

| **S. No** | **Chemical Compound** | **RT** | **Molecular weight** | **Molecular Formula** | **Area %** |
| --- | --- | --- | --- | --- | --- |
| 1 | Benzyl alcohol | 7.838 | 108.13 | C_7_H_8_O | 0.26 |
| 2 | 1H-Pyrazole, 4,5-dihydro-5,5-dimethyl-4-isopropylidene | 9.122 | 98.14 | C_5_H_10_N_2_ | 0.96 |
| 3 | Ethanol, 2-(2-butoxyethoxy)- | 9.659 | 162.22 | C_8_H_18_O_3_ | 1.24 |
| 4 | Benzoic acid | 10.019 | 122.12 | C_7_H_6_O_2_ | 0.43 |
| 5 | 2-Dodecene, (Z)- | 10.942 | 168.31 | C_12_H_24_ | 0.71 |
| 6 | 2-Piperidinone | 11.252 | 99.13 | C_5_H_9_NO | 1.09 |
| 7 | 2-Coumaranone | 11.697 | 134.13 | C_8_H_6_O_2_ | 0.50 |
| 8 | Indole | 12.410 | 117.14 | C_8_H_7_N | 0.24 |
| 9 | 9-Octadecene, (E)- | 13.492 | **128.17** | C_13_H_23_ClO_2_ | 2.08 |
| 10 | Phenol, 2,4-bis (1,1-dimethylethyl | 13.786 | 135.16 | C_8_H_9_NO | 3.19 |
| 11 | Toluene | 14.214 | 151.16 | C_8_H_9_NO_2_ | 0.71 |
| 12 | Acetamide, N-(2-phenylethyl)- | 15.455 | 280.5316 | C_20_H_40_ | 1.16 |
| 13 | E-14-Hexadecenal | 15.791 | **256.42** | C_16_H_32_O_2_ | 3.11 |
| 14 | Heneicosane | 16.462 | **278.34** | C_16_H_22_O_4_ | 0.44 |
| 15 | Benzophenone | 16.655 | 150.13 | C_8_H_6_O_3_ | 0.25 |
| 16 | Cyclotetradecane | 17.443 | 278.34 | C_16_H_22_O_4_ | 0.84 |
| 17 | Benzo[h]quinoline, 2,4-dimethyl- | 17.628 | **334.45** | C_20_H_30_O4 | 0.41 |
| 18 | 5-Eicosene, (E)- | 17.871 | **334.45** | C_20_H_30_O_4_ | 2.55 |
| 19 | 1,2-Benzenedicarboxylic acid, bis(1-methylethyl) ester | 18.467 | **250.29** | C_14_H_18_O_4_ | 7.71 |
| 20 | 2-Amino-4,5-dimethylthiazole | 18.685 | 311.5 | C_16_H_33_NO | 0.54 |
| 21 | 3-Pyrrolidin-2-yl-propionic acid | 18.819 | 336.63 | C_24_H_48_ | 0.89 |
| 22 | Dibutyl phthalate | 19.020 | 238.41 | C_16_H_30_O | 8.19 |
| 23 | 5-Acetoxy-4-oxohex-2-enoic acid, methyl ester | 19.205 | 149.23 | C_10_H_15_N | 0.49 |
| 24 | Di-sec-butyl phthalate | 19.557 | **444.12** | C_11_H_20_Br_2_ | 10.94 |
| 25 | N-Acetyltyramine | 19.764 | **174.24** | C_11_H_14_N_2_ | 12.20 |
| 26 | 2,3-Dimethyl-1-hexene | 19.966 | 536.00 | C_18_H_37_NO | 3.06 |
| 27 | Octadecanophenone | 20.329 | **136.19** | C_9_H_12_O | 4.97 |
| 28 | Pyrrolo[1,2-a]pyrazine-1,4-dione, hexahydro-3-(2-methylpropyl)- | 20.799 | **536.95** | C_36_H_72_O_2_ | 5.49 |
| 29 | 1-(2-Methylallyl)azetidine | 21.268 | **358.55** | C_21_H_42_O _4_ | 0.92 |
| 30 | Heptadecyl heptafluorobutyrate | 21.529 | **390.56** | C_24_H_38_O_4_ | 0.92 |
| 31 | Cycloundecane, 1,1,2-trimethyl- | 21.730 | 144.17 | C_9_H_8_N_2_ | 0.29 |
| 32 | Disparlure | 21.898 | **309.53** | C_20_H_39_NO | 0.58 |
| 33 | Cyclopentane, hexyl | 22.107 | **285.49** | C_18_H_36_O_3_ | 0.27 |
| 34 | Tricosane | 22.334 | 212.28 | C_12_H_20_O_3_ | 0.34 |
| 35 | 4-Chlorobutyric acid, 2-biphenyl ester | 23.231 | **285.49** | C_18_H_36_O_3_ | 3.69 |
| 36 | 2,5-Piperazinedione, 3,6-bis(2-methylpropyl)- | 23.391 | 212.28 | C_12_H_20_O_3_ | 2.48 |
| 37 | 5,8-Tridecadione | 23.567 | **285.49** | C_18_H_36_O_3_ | 0.31 |
| 38 | Fumaric acid, monoamide, N-benzyl-N-phenylethyl-, ethyl ester | 23.919 | 212.28 | C_12_H_20_O_3_ | 1.74 |
| 39 | Diisooctyl phthalate | 24.146 | **285.49** | C_18_H_36_O_3_ | 3.76 |
| 40 | Cyclo-(l-leucyl-l-phenylalanyl) | 25.532 | 212.28 | C_12_H_20_O_3_ | 0.29 |
| 41 | Cyclopentathiazole | 25.010 | 212.28 | C_12_H_20_O_3_ | 4.17 |
| 42 | 2(1H)-Pyrimidinone, 4-amino-5-methyl- | 25.228 | **285.49** | C_18_H_36_O_3_ | 1.62 |
| 43 | Squalene | 25.421 | 212.28 | C_12_H_20_O_3_ | 1.27 |
| 44 | 13-Docosenamide, (Z)- | 26.142 | **285.49** | C_18_H_36_O_3_ | 2.18 |
| 45 | 1-Nitro-bicyclo[6.1.0]nonan-2-one | 26.327 | 212.28 | C_12_H_20_O_3_ | 0.46 |

**Table S2. Potential BGCs in Streptomyces sp. VITGV156, predicted using antiSMASH 6.0 (descending order). Their scaffolds, clusters and percentage resemblance are given.**

| **S. No.** | **Scaffolds** | **Nature of Cluster** | **Corresponding known cluster** | **Resemblance (%)** |
| --- | --- | --- | --- | --- |
| 1 | Core hits - 17.2 | Terpene | Geosmin | 100% |
| 2 | Core hits - 7.1 | Ectoine | Ectoine | 100% |
| 3 | Core hits - 12.1 | Terpene | Albaflavenone | 100% |
| 4 | Core hits - 18.2 | Terpene | Hopene | 100% |
| 5 | Core hits - 21.4 | NRPS | Coelichelin | 100% |
| 6 | Core hits - 21.6 | Lanthipeptide-class-iii | SapB | 100% |
| 7 | Core hits - 3.1 | NRPS-like | Streptothricin | 95% |
| 8 | Core hits - 21.5 | NRPS, lanthipeptide-class-i | Coelibactin | 90% |
| 9 | Core hits - 7.3 | Siderophore | Desferrioxamine B / Desferrioxamine E | 83% |
| 10 | Core hits - 18.1 | NRPS | CDA1b / CDA2a / CDA2b / CDA3a / CDA3b / CDA4a / CDA4b | 72% |
| 11 | Core hits - 13.1 | T2PKS, PKS-like | Fluostatins M-Q | 67% |
| 12 | Core hits - 12.2 | T2PKS | Spore Pigment | 66% |
| 13 | Core hits - 2.2 | Terpene | Isorenieratene | 63% |
| 14 | Core hits - 7.2 | Melanin | Melanin | 60% |
| 15 | Core hits - 18.3 | T1PKS | Vicenistatin | 60% |
| 16 | Core hits - 11.1 | Lanthipeptide-class-III | Catenulipeptin | 60% |
| 17 | Core hits - 21.3 | RiPP-like | Informatipeptin | 42% |
| 18 | Core hits - 21.1 | T1PKS | Streptovaricin | 29% |
| 19 | Core hits - 20.1 | T1PKS | Nystatin A1 | 27% |
| 20 | Core hits - 2.1 | Indole | 5-isoprenylindole-3-carboxylate β-D-Glycosyl ester | 23% |
| 21 | Core hits - 19.1 | T1PKS | Sipanmycin | 13% |
| 22 | Core hits - 10.2 | PKS-like, furan, Lanthipeptide-class-V | Methylenomycin A | 9% |
| 23 | Core hits - 17.3 | Siderophore | Paulomycin | 9% |
| 24 | Core hits - 3.2 | T3PKS | Herboxidiene | 8% |
| 25 | Core hits -14.1 | Butyrolactone | Prejadomycin / Rabelomycin / Gaudimycin C / Gaudimycin D / Uwm6 / Gaudimycin A | 6% |
| 26 | Core hits - 21.2 | Terpene | Versipelostatin | 5% |
| 27 | Core hits - 10.1 | NRPS-like | - | - |
| 28 | Core hits - 16.1 | Siderophore | - | - |
| 29 | Core hits - 17.1 | RiPP-like | - | - |

Putative gene clusters coding for secondary metabolites in *Streptomyces* sp. VITGV156

Non – Ribosomal Peptide Synthetase - NRPS

Type 1 Polyketide Synthases - T1PKS

RiPP - Ribosomal Synthesized and Post-translationally modified Peptides

**Table S3** Compounds obeying Lipinski’s rule of 5 of the Compounds of *Streptomyces* sp. VITGV156

| **S.No** | **Chemical compound** | **Lipinski’s rule of 5/ No. of violations** | **Molecular weight** | **Log P** | **H-bond donor** | **H-bond acceptor** | **Molecular refractivity** |
| --- | --- | --- | --- | --- | --- | --- | --- |
|  | Benzyl alcohol | Yes; 0 | 108.13 | 1.128 | 1 | 1 | 32.57 |
|  | 1H-Pyrazole, 4,5-dihydro-5,5-dimethyl-4-isopropylidene | Yes; 0 | 98.14 | 1.794 | 1 | 2 | 51.75 |
|  | Ethanol, 2-(2-butoxyethoxy)- | Yes; 0 | 162.22 | 0.727 | 1 | 3 | 43.90 |
|  | Benzoic acid | Yes; 0 | 122.12 | 1.957 | 1 | 2 | 33.40 |
|  | 2-Dodecene, (Z)- | Yes; 1 | 168.31 | 6.143 | 0 | 0 | 59.32 |
|  | 2-Piperidinone | Yes; 0 | 99.13 | -0.278 | 1 | 2 | 30.95 |
|  | 2-Coumaranone | Yes; 0 | 134.13 | 2.239 | 1 | 2 | 35.99 |
|  | 5H-1-Pyrindine | Yes; 0 | 117.15 | 1.376 | 0 | 1 | 37.18 |
|  | 9-Octadecene, (E)- | Yes; 1 | **128.171** | 8.719 | 0 | 0 | 88.17 |
|  | Phenol, 2,4-bis (1,1-dimethylethyl | Yes; 1 | 135.1632 | 4.832 | 1 | 1 | 88.17 |
|  | Toluene | Yes; 0 | 151.16 | 2.544 | 0 | 0 | 31.41 |
|  | Acetamide, N-(2-phenylethyl)- | Yes; 0 | 280.5316 | 1.098 | 1 | 2 | 48.83 |
|  | E-14-Hexadecenal | Yes; 1 | **256.4241** | 6.087 | 0 | 1 | 78.75 |
|  | Heneicosane | Yes; 1 | **278.3435** | 10.427 | 0 | 0 | 103.06 |
|  | Benzophenone | Yes; 0 | 150.13 | 3.073 | 0 | 1 | 56.32 |
|  | Cyclotetradecane | Yes; 1 | 278.3435 | 7.412 | 0 | 0 | 67.30 |
|  | Benzo[h]quinoline, 2,4-dimethyl- | Yes; 0 | 334.456 | 4.233 | 0 | 1 | 69.18 |
|  | 1-Octadecene | Yes; 1 | **252.5** | 8.871 | 0 | 0 | 88.17 |
|  | 1,2-Benzenedicarboxylic acid, bis(1-methylethyl) ester | Yes; 0 | **250.2903** | 3.163 | 0 | 4 | 68.23 |
|  | 2-Amino-4,5-dimethylthiazole | Yes; 0 | 311.5 | 0.716 | 2 | 2 | 36.45 |
|  | 2-Acetylpyrrolidine | Yes; 0 | 113.16 | -0.21 | 1 | 2 | 35.76 |
|  | 2-(Heptyloxycarbonyl)benzoic acid | Yes; 0 | 264.32 | 4.161 | 1 | 4 | 73.52 |
|  | 5-Acetoxy-4-oxohex-2-enoic acid, methyl ester | Yes; 0 | 149.233 | 0.563 | 0 | 5 | 47.67 |
|  | Di-sec-butyl phthalate | Yes; 0 | **444.12** | 4.019 | 0 | 4 | 77.84 |
|  | Tyramine, N-formyl- | Yes; 0 | 165.19 | 0.559 | 2 | 3 | 46.44 |
|  | 2,3-Dimethyl-1-hexene | Yes; 0 | 536.0 | 3.859 | 0 | 0 | 40.10 |
|  | Octadecanophenone | Yes; 1 | **136.19100** | 9.064 | 0 | 1 | 113.55 |
|  | Phenol, 3,5-dimethoxy- | Yes; 0 | 154.16 | 1.701 | 1 | 3 | 41.45 |
|  | 1-(2-Methylallyl)azetidine | Yes; 0 | **358.5558** | 1.535 | 0 | 1 | 39.99 |
|  | Heptadecyl heptafluorobutyrate | Yes; 1 | **390.56** | 8.976 | 0 | 2 | 104.82 |
|  | Cycloundecane, 1,1,2-trimethyl- | Yes; 1 | 144.17 | 7.054 | 0 | 0 | 67.04 |
|  | Disparlure | Yes; 1 | **309.53** | 8.033 | 0 | 1 | 92.42 |
|  | Cyclopentane, hexyl | Yes; 1 | **285.49** | 5.723 | 0 | 0 | 52.88 |
|  | Tricosane | Yes; 1 | 212.28 | 11.214 | 0 | 0 | 112.67 |
|  | 4-Chlorobutyric acid, 2-biphenyl ester | Yes; 1 | **285.49** | 3.949 | 0 | 2 | 77.79 |
|  | Azacyclohexan-3-one, 1-tert-butyl- | Yes; 0 | 155.24 | 0.988 | 0 | 2 | 50.31 |
|  | 5,8-Tridecadione | Yes; 0 | **285.49** | 2.934 | 0 | 2 | 65.00 |
|  | Fumaric acid, monoamide, N-benzyl-N-phenylethyl-, ethyl ester | Yes; 0 | 212.28 | 3.679 | 0 | 4 | 98.26 |
|  | Diisooctyl phthalate | Yes; 1 | **285.49** | 7.494 | 0 | 4 | 116.30 |
|  | 2,5-Piperazinedione, 3-(hydroxymethyl)-6-(phenylmethyl)- | Yes; 0 | 234.25 | 0.379 | 3 | 5 | 68.32 |
|  | Cyclopentathiazole | Yes; 0 | 212.28 | 0.547 | 2 | 2 | 39.14 |
|  | 2(1H)-Pyrimidinone, 4-amino-5-methyl- | Yes; 0 | **285.49** | -1.317 | 3 | 4 | 34.23 |
|  | Squalene | Yes; 1 | 212.28 | 12.248 | 0 | 0 | 143.48 |
|  | 9-Octadecenamide, (Z)- | Yes; 1 | 281.5 | 6.443 | 2 | 2 | 91.07 |
|  | 1-Nitro-bicyclo[6.1.0]nonan-2-one | Yes; 0 | 212.28 | 1.566 | 0 | 4 | 49.49 |

**Table S4** Binding affinity of *Streptomyces* sp. VITGV156. The highest binding score value was highlighted.

| **S. No** | **Chemical Compound** | **Pubchem ID** | **Binding Energy (Kcal/mol)** | |
| --- | --- | --- | --- | --- |
|  |  |  | **5m18** | **6nvu** |
|  | Benzyl alcohol | 244 | -4.9 | -4.7 |
|  | 1H-Pyrazole, 4,5-dihydro-5,5-dimethyl-4-isopropylidene | 557693 | -5.0 | -4.9 |
|  | Ethanol, 2-(2-butoxyethoxy)- | 8177 | -4.3 | -4.2 |
|  | Benzoic acid | 243 | -5.7 | -5.1 |
|  | 2-Dodecene, (Z)- | 5364558 | -5.2 | -4.5 |
|  | 2-Piperidinone | 12665 | -4.3 | -4.4 |
|  | 2-Coumaranone | 68382 | -5.6 | -5.8 |
|  | 5H-1-Pyrindine | 575987 | -5.4 | -4.9 |
|  | 9-Octadecene, (E)- | 5364599 | -5.1 | -4.0 |
|  | Phenol, 2,4-bis (1,1-dimethylethyl | 7311 | -7.0 | -6.3 |
|  | Toluene | 1140 | -4.6 | -4.4 |
|  | Acetamide, N-(2-phenylethyl)- | 70143 | -5.6 | -5.3 |
|  | E-14-Hexadecenal | 5363106 | -5.4 | -4.4 |
|  | Heneicosane | 12403 | -5.1 | -3.9 |
|  | Benzophenone | 3102 | -6.7 | -6.3 |
|  | Cyclotetradecane | 67524 | -6.0 | -5.1 |
|  | Benzo[h]quinoline, 2,4-dimethyl- | 610182 | -7.5 | -7.2 |
|  | 1-Octadecene | 8217 | -5.2 | -4.1 |
|  | 1,2-Benzenedicarboxylic acid, bis(1-methylethyl) ester | 11799 | -6.0 | -5.8 |
|  | 2-Amino-4,5-dimethylthiazole | 73238 | -4.0 | -4.2 |
|  | 2-Acetylpyrrolidine | 550747 | -4.5 | -4.2 |
|  | 2-(Heptyloxycarbonyl)benzoic acid | 111740 | -6.2 | -5.5 |
|  | 5-Acetoxy-4-oxohex-2-enoic acid, methyl ester | 5363724 | -5.3 | -5.2 |
|  | Di-sec-butyl phthalate | 249496 | -6.2 | -5.6 |
|  | Tyramine, N-formyl- | 577647 | -5.5 | -5.2 |
|  | 2,3-Dimethyl-1-hexene | 86061 | -4.6 | -4.4 |
|  | Octadecanophenone | 81244 | -5.3 | -4.6 |
|  | Phenol, 3,5-dimethoxy- | 10383 | -4.9 | -4.7 |
|  | 1-(2-Methylallyl)azetidine | 550700 | -3.9 | -3.9 |
|  | Heptadecyl heptafluorobutyrate | 545577 | -5.9 | -4.6 |
|  | Cycloundecane, 1,1,2-trimethyl- | 544748 | -6.8 | -5.7 |
|  | Disparlure | 205983 | -5.3 | -4.3 |
|  | Cyclopentane, hexyl | 138257 | -4.8 | -4.2 |
|  | Tricosane | 12534 | -5.4 | -4.3 |
|  | 4-Chlorobutyric acid, 2-biphenyl ester | 54792714 | -6.3 | -6.3 |
|  | Azacyclohexan-3-one, 1-tert-butyl- | 559313 | -5.5 | -5.0 |
|  | 5,8-Tridecadione | 538300 | -4.9 | -4.2 |
|  | Fumaric acid, monoamide, N-benzyl-N-phenylethyl-, ethyl ester | 91699612 | -7.2 | -6.6 |
|  | Diisooctyl phthalate | 33934 | -6.0 | -5.7 |
|  | 2,5-Piperazinedione, 3-(hydroxymethyl)-6-(phenylmethyl)- | 563315 | -6.9 | -6.8 |
|  | Cyclopentathiazole | 332255 | -4.6 | -4.5 |
|  | 2(1H)-Pyrimidinone, 4-amino-5-methyl- | 65040 | -4.9 | -4.9 |
|  | Squalene | 638072 | -7.3 | -4.8 |
|  | 9-Octadecenamide, (Z)- | 5283387 | -5.6 | -5.0 |
|  | 1-Nitro-bicyclo[6.1.0]nonan-2-one | 548951 | -6.8 | -5.9 |

**Table S5** Toxicity analysis of Streptomyces sp. VITGV156

| **S.No** | **Chemical compound** | **Mutagenic** | **Tumorigenic** | **Irritant** | **Reproductive effective** | **Drug likeness** |
| --- | --- | --- | --- | --- | --- | --- |
|  | Benzyl alcohol | Red | Red | Red | Red | -2.03 |
|  | 1H-Pyrazole, 4,5-dihydro-5,5-dimethyl-4-isopropylidene | Green | Green | Green | Green | 2.77 |
|  | Ethanol, 2-(2-butoxyethoxy)- | Green | Green | Red | Yellow | -26.88 |
|  | Benzoic acid | Red | Green | Red | Green | -1.4 |
|  | 2-Dodecene, (Z)- | Green | Green | Green | Green | -23.49 |
|  | 2-Piperidinone | Green | Green | Green | Green | -3.66 |
|  | 2-Coumaranone | Green | Green | Green | Green | -1.06 |
|  | 5H-1-Pyrindine | Green | Green | Green | Green | -2.76 |
|  | 9-Octadecene, (E)- | Green | Green | Green | Green | -24.05 |
|  | Phenol, 2,4-bis (1,1-dimethylethyl | Green | Green | Green | Green | -5.83 |
|  | Toluene | Red | Red | Red | Red | -2.47 |
|  | Acetamide, N-(2-phenylethyl)- | Green | Green | Green | Green | 2.82 |
|  | E-14-Hexadecenal | Red | Green | Red | Red | -12.82 |
|  | Heneicosane | Green | Green | Green | Green | -20.4 |
|  | Benzophenone | Red | Red | Green | Green | -0.72 |
|  | Cyclotetradecane | Green | Green | Green | Green | -7.63 |
|  | Benzo[h]quinoline, 2,4-dimethyl- | Yellow | Yellow | Green | Green | -3.84 |
|  | 1-Octadecene | Green | Green | Red | Green | -27.66 |
|  | 1,2-Benzenedicarboxylic acid, bis(1-methylethyl) ester | Red | Yellow | Yellow | Red | -11.42 |
|  | 2-Amino-4,5-dimethylthiazole | Green | Green | Green | Green | -1.05 |
|  | 2-Acetylpyrrolidine | Green | Green | Green | Red | -1.12 |
|  | 2-(Heptyloxycarbonyl)benzoic acid | Red | Yellow | Yellow | Red | -26.76 |
|  | 5-Acetoxy-4-oxohex-2-enoic acid, methyl ester | Green | Green | Red | Green | -5.97 |
|  | Di-sec-butyl phthalate | Red | Yellow | Yellow | Red | -9.91 |
|  | Tyramine, N-formyl- | Green | Green | Green | Green | 1.02 |
|  | 2,3-Dimethyl-1-hexene | Green | Green | Red | Green | -16.7 |
|  | Octadecanophenone | Green | Green | Green | Green | -22.42 |
|  | Phenol, 3,5-dimethoxy- | Green | Green | Green | Green | -10.9 |
|  | 1-(2-Methylallyl) azetidine | Green | Green | Green | Green | -6.29 |
|  | Heptadecyl heptafluorobutyrate | Red | Green | Yellow | Green | -122.9 |
|  | Cycloundecane, 1,1,2-trimethyl- | Green | Green | Red | Green | -7.64 |
|  | Disparlure | Green | Yellow | Green | Green | -20.12 |
|  | Cyclopentane, hexyl | Green | Green | Green | Green | -15.3 |
|  | Tricosane | Green | Green | Green | Green | -20.4 |
|  | 4-Chlorobutyric acid, 2-biphenyl ester | Yellow | Red | Green | Red | -9.64 |
|  | Azacyclohexan-3-one, 1-tert-butyl- | Green | Green | Green | Green | 1.57 |
|  | 5,8-Tridecadione | Green | Green | Red | Green | -12.38 |
|  | Fumaric acid, monoamide, N-benzyl-N-phenylethyl-, ethyl ester | Green | Green | Red | Green | -6.39 |
|  | Diisooctyl phthalate | Red | Yellow | Yellow | Red | -19.4 |
|  | 2,5-Piperazinedione, 3-(hydroxymethyl)-6-(phenylmethyl)- | Green | Green | Green | Green | 6.12 |
|  | Cyclopentathiazole | Green | Green | Green | Green | -1.08 |
|  | 2(1H)-Pyrimidinone, 4-amino-5-methyl- | Green | Green | Green | Green | 1.23 |
|  | Squalene | Green | Green | Green | Green | -3.52 |
|  | 9-Octadecenamide, (Z)- | Green | Green | Green | Green | -27.66 |
|  | 1-Nitro-bicyclo[6.1.0]nonan-2-one | Green | Green | Green | Green | -13.2 |

**Table S6** Biological activity of *Streptomyces* sp. VITGV156

| **S.No** | **Compounds** | **Antibacterial Activity (Pa-value)** | **Antifungal Activity (Pa- value)** | **Antibiotic Activity (Pa-value)** |
| --- | --- | --- | --- | --- |
|  | Benzyl alcohol | 0.252 | 0.137 | 0.336 |
|  | 1H-Pyrazole, 4,5-dihydro-5,5-dimethyl-4-isopropylidene | 0.188 | - | 0.019 |
|  | Ethanol, 2-(2-butoxyethoxy)- | 0.238 | 0.380 | 0.107 |
|  | Benzoic acid | 0.362 | 0.329 | 0.194 |
|  | 2-Dodecene, (Z)- | 0.383 | 0.530 | 0.190 |
|  | 2-Piperidinone | 0.187 | 0.208 | 0.110 |
|  | 2-Coumaranone | 0.233 | 0.385 | 0.110 |
|  | 5H-1-Pyrindine | 0.166 | 0.174 | 0.109 |
|  | 9-Octadecene, (E)- | 0.320 | 0.471 | 0.159 |
|  | Phenol, 2,4-bis (1,1-dimethylethyl | 0.206 | 0.356 | 0.093 |
|  | Toluene | 0.248 | 0.316 | 0.135 |
|  | Acetamide, N-(2-phenylethyl)- | 0.173 | - | 0.021 |
|  | E-14-Hexadecenal | 0.447 | 0.595 | 0.208 |
|  | Heneicosane | 0.287 | 0.377 | 0.151 |
|  | Benzophenone | 0.199 | 0.291 | 0.025 |
|  | Cyclotetradecane | 0.267 | 0.301 | 0.157 |
|  | Benzo[h]quinoline, 2,4-dimethyl- | 0.137 | 0.203 | - |
|  | 1-Octadecene | 0.353 | 0.535 | 0.158 |
|  | 1,2-Benzenedicarboxylic acid, bis(1-methylethyl) ester | 0.274 | 0.400 | 0.144 |
|  | 2-Amino-4,5-dimethylthiazole | 0.414 | 0.176 | 0.178 |
|  | 2-Acetylpyrrolidine | 0.269 | - | 0.131 |
|  | 2-(Heptyloxycarbonyl)benzoic acid | 0.308 | 0.424 | 0.128 |
|  | 5-Acetoxy-4-oxohex-2-enoic acid, methyl ester | 0.327 | 0.560 | 0.202 |
|  | Di-sec-butyl phthalate | 0.267 | 0.387 | 0.145 |
|  | Tyramine, N-formyl- | 0.233 | 0.254 | 0.096 |
|  | 2,3-Dimethyl-1-hexene | 0.317 | 0.436 | 0.118 |
|  | Octadecanophenone | 0.235 | 0.403 | 0.098 |
|  | Phenol, 3,5-dimethoxy- | 0.302 | 0.393 | 0.160 |
|  | 1-(2-Methylallyl)azetidine | 0.165 | 0.407 | 0.014 |
|  | Heptadecyl heptafluorobutyrate | 0.126 | - | - |
|  | Cycloundecane, 1,1,2-trimethyl- | 0.322 | 0.343 | 0.168 |
|  | Disparlure | 0.395 | 0.565 | 0.203 |
|  | Cyclopentane, hexyl | 0.259 | 0.413 | 0.125 |
|  | Tricosane | 0.287 | 0.377 | 0.151 |
|  | 4-Chlorobutyric acid, 2-biphenyl ester | - | 0.340 | 0.119 |
|  | Azacyclohexan-3-one, 1-tert-butyl- | 0.178 | - | 0.104 |
|  | 5,8-Tridecadione | 0.262 | 0.476 | 0.139 |
|  | Fumaric acid, monoamide, N-benzyl-N-phenylethyl-, ethyl ester | - | 0.237 | - |
|  | Diisooctyl phthalate | 0.233 | 0.426 | 0.092 |
|  | 2,5-Piperazinedione, 3-(hydroxymethyl)-6-(phenylmethyl)- | 0.293 | 0.436 | 0.155 |
|  | Cyclopentathiazole | 0.284 | 0.381 | 0.125 |
|  | 2(1H)-Pyrimidinone, 4-amino-5-methyl- | 0.166 | 0.065 | 0.024 |
|  | Squalene | 0.397 | 0.531 | 0.194 |
|  | 9-Octadecenamide, (Z)- | 0.311 | 0.448 | 0.148 |
|  | 1-Nitro-bicyclo[6.1.0]nonan-2-one | 0.197 | 0.196 | - |

**
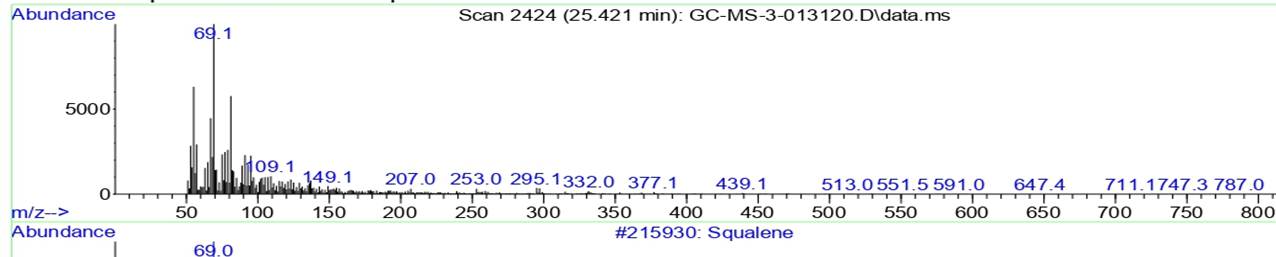
Fig. S1.** **GC-MS chromatogram showing the presence of Squalene in crude extracts of *Streptomyces* sps. VITGV156**
